# A Theory of Everything: Overlapping Neurobiological Mechanisms of Psychotherapies of Fear and Anxiety Related Disorders

**DOI:** 10.3389/fnbeh.2018.00328

**Published:** 2019-01-08

**Authors:** Arash Javanbakht

**Affiliations:** Department of Psychiatry and Behavioral Neurosciences, Wayne State University School of Medicine, Detroit, MI, United States

**Keywords:** psychotherapy, neurobiology, neuroscience, psychoanalysis, cognitive therapy, exposure therapy, anxiety, PTSD

## Abstract

Similarities within the phenomenology, neurobiology, psychotherapeutic, and pharmacological treatments of distinctly categorized anxiety and fear related disorders suggest the involvement of common neurobiological mechanisms in their formation. This theory of integration is the focus of the Research Domain Criteria (RDoC) approach initiated by the NIH. The current article explores potential facets of overlap among mainstream methods of psychotherapy for anxiety, fear, and trauma related disorders. These overlaps include associative learning of safety, cognitive reappraisal and emotion regulation, therapist as a social safety cue, and contextualization. Temporal contextualization and placing memories in their time and place will be suggested as a potentially important, and less explored aspect of psychotherapy.

## Introduction

As our understanding of the neurobiology of mental processes and disorders solidifies, we are beginning to attempt theory integration across phenomenologically and categorically distinct psychiatric disorders. This new approach aims to examine the similarities of these disorders through the lens of specific mental functions. The hallmark of this new direction in neuroscience is Research Domain Criteria (RDoC), which aims to “develop, for research purposes, new ways of classifying mental disorders based on behavioral dimensions and neurobiological measures” (Insel et al., 2010). Discussion of similarities between seemingly distinct mental disorders has been present in psychiatry since the days of Freud. Defense mechanisms, for example, were developed to explain a plethora of mental illnesses including anxiety disorders, depression, and somatoform disorders (Cramer, 2015). Similarly, psychoanalysis, psychodynamic psychotherapy, cognitive therapy, and behavioral therapy, are offered to treat a wide range of anxiety, fear, and trauma related disorders, with only minor adjustments (Butler et al., 2006). When introduced to the armamentarium of psychiatric treatments, psychopharmacology also provided evidence of similar treatment across these diagnoses. After decades, Serotonin Reuptake Inhibitors (SSRIs), and Serotonin Norepinephrine Reuptake Inhibitors (SNRIs) are still the mainstream treatments for all of the anxiety disorders, posttraumatic stress disorder (PTSD), Obsessive Compulsive Disorder (OCD), and depressive disorders, with similar efficacy across the board (Finley, 1994). Our current neurobiological understanding of anxiety and trauma related disorders is still virtually indistinguishable. In the majority of these disorders the main areas of anatomical and functional significance are the medial PFC (mPFC), the dorsolateral prefrontal cortex (dlPFC), the anterior cingulate cortex (ACC), the insula, the hippocampus, and the amygdala (Duval et al., 2015). One could argue that these similarities are the product of the lack of precision our current methods in neuroscience provide. This could explain why the current general treatment for anxiety disorders is not very effective- for example; SSRIs are only moderately more effective than placebo in treatment of anxiety and depressive disorders (Goodnick and Goldstein, 1998). One could also argue (without the intent of being mutually exclusive) that there may be similar mechanisms, both biological and psychological, involved in formation of seemingly different anxiety disorders.

In this article, considering both the immense foundation of contemporary psychology and latest findings in neurobiology of anxiety disorders and trauma, I suggest multiple facets of overlap between the seemingly distinct mainstream psychotherapeutic methods for the treatment of fear, anxiety, and trauma. I will then discuss the use of these mechanisms within each method of psychotherapy, and the clinical implication. The intention of this work is not to fully explain the neurobiology of psychotherapy, but to discuss the commonalities between the mainstream methods.

## Associative Learning of Fear and Safety, Stimulus Generalization, Cognitive Schemas, and Transferences

Associative learning is a common method used across species to make sense of the world. The most commonly studied form of associative learning is Pavlovian fear conditioning, during which a neutral cue (e.g., a triangle) is paired with an inherently aversive stimulus (e.g., a shock), repeatedly (Milad and Quirk, 2012). After the training phase, which consists of this repeated pairing, the organism learns that the previously neutral cue is a predictor of the aversive stimulus. As a result, the conditioned stimulus invokes the same fear response as the aversive stimulus. Interestingly, the brain areas involved in associative fear learning (mainly amygdala, insula, ACC, and hippocampus) largely overlap with those involved in psychopathology of anxiety disorders and PTSD (Greco and Liberzon, 2016). Similarly, appetitive conditioning creates an associative learning between a neutral cue and an inherently appetitive stimulus (e.g., food; Martin-Soelch et al., 2007). In humans, associative learning can take place by personal experience, social observation, or verbal information relayed through instruction (Olsson and Phelps, 2007). For instance, one can be afraid of a dog by being personally attacked, by seeing another person attacked, by being told the dog is dangerous, or by reading a sign that says, “beware of dog.” Associative learning can occur with simple cues (e.g., a triangle), social cues (e.g., a picture of a face), or a more complex combination of perceptual inputs, namely context (contextual conditioning; Rudy et al., 2004). During contextual conditioning, an aversive stimulus is paired with a physical environment rather than a specific cue. However, it is suggested that context is not only physical environment, but social, temporal, internal, and cognitive input as well (Maren et al., 2013). Cognitive information, acting as a vital element of learning in humans, seems to function similarly to physical context in fear and safety learning (Javanbakht et al., 2017).

Whether one agrees to the broader definition of context or not, it is conceivable that a combination of physical, temporal, and cognitive cues could be paired with an aversive or appetitive response. For instance, both words “red” and “car” could be paired with an aversive experience separately, as could the combination “red car” in creating a fear response to a “red car.” Clinically, a person hit by a red car could develop a fear of cars, driving, red cars, or even proximity to roads where cars are seen.

More complex cognitive constructs (cognitive schemas or distortions in cognitive therapy) can similarly be paired with an aversive or appetitive response. According to cognitive theory, cognitive schemas are organized patterns of thoughts (Piaget, 1950), while cognitive distortions (Beck, 1963, 2008) are biased cognitive concepts. Similar to associative learning, schemas can form by personal experience similar to experiential conditioning (red car driven by a young man hit me last year => view of a red car, or a red car driven by a young man trigger the emotion of fear), social observation (red car driven by a young man hit my neighbor last year), or verbal information (I heard that the young man with the red car in the neighborhood is a careless driver). All these seemingly different inputs can evoke a fear response at perception, imagination, or news of the young neighbor driving a red car. Such learning can be then generalized to other similar conditions, leading to the expansion of a cognitive schema.

Associative learning of fear can be generalized to perceptually, conceptually, or cognitively similar cues (Dunsmoor and Paz, 2015). Stimulus generalization is a process through which associative learning extends to new stimuli that are related in some way to the stimulus originally associated with an emotional response. Fear generalization is shown to positively correlate with fear intensity, suggesting anxiety’s crucial role in fear generalization (Dunsmoor et al., 2009). Generalization is an adaptive response that helps organisms survive by avoiding relatively similar threats. For example, if one was attacked by a black bear, generalized fear of brown bears is reasonable in respect of avoiding a future attack. In an early work on stimulus generalization, Guttman and Kalish (1956) showed that pigeons can generalize the associative learning response to colors of light with nearly similar wavelengths to the light used in associative learning. Human studies have also shown generalization of fear response to shapes close in size to the conditioned cue (Lissek et al., 2008). This form of learning seems to be involved in development of phobias: one can become afraid of all breeds of dogs after being attacked by a single breed of dog. While stimulus generalization can occur to physical sensory aspects of the stimulus, tone of sound, or even facial structure in humans (Honig and Urcuioli, 1981), recent research supports possibility of generalization of associative learning to conceptually or semantically similar stimuli (Maltzman, 1977). In an interesting study, Dunsmoor et al. (2011) showed that fear generalization was stronger in a group of participants that had learned association between two conceptually similar words, compared to the groups who learned the same association between unrelated or mismatched words.

At a more complex level, generalization of fear may occur to more abstract cognitive schemas or social constructs, such as the concept of “authority.” Such conceptual and symbolic generalization has been a core focus of psychoanalysis and psychodynamic theory. For example, a person frequently mistreated by a parental figure during childhood, may generalize a fear of authority figures, and “*transfer*” the same response onto a similar relational pattern (Javanbakht and Ragan, 2008). *Transference* is a process during which one transfers emotions that were experienced in the past, to a conceptually or socially similar context in the here and now. Transference can be explained as a form of generalized fear or other emotions to a current social cue, similar to those of the past. In other words, an emotional response is linked to a specific social, cognitive, or conceptual pattern.

In summary, associative learning can occur with internal and external stimuli, a variety of simple cues, social cues, social context, cognitive context, and more abstract cognitive concepts such as schemas, cognitive distortions, and transferences by a relatively similar mechanism.

In *Extinction Learning*, it is learned that a previously conditioned stimulus is not associated with an aversive experience anymore. In the laboratory during extinction learning, the conditioned stimulus is repeatedly presented in the absence of the aversive stimulus, leading to extinction of the fear response (Milad and Quirk, 2012). Importantly, extinction learning is not simply erasure of the learned fear, but rather an additional learning that the conditioned stimulus is safe in the new physical/temporal/social context; for that reason, the extinguished fear can return (Milad et al., 2005). Similar to fear, extinction learning can take place *via* personal experience, social observation, and cognitive instruction (Koenig and Henriksen, 2005; Javanbakht et al., 2017). Contemporary laboratory models of exposure therapy, a mainstream treatment of fear related disorders, are based on extinction learning (Abramowitz, 2013). The brain areas that are involved in retention of extinction learning (vmPFC, dlPFC, and hippocampus) commonly show impaired anatomy and function in anxiety and trauma related disorders (Duval et al., 2015; Greco and Liberzon, 2016). The same areas are involved in the cognitive modulation of fear responses, such as in reappraisal. In laboratory, during reappraisal, effortful change of the meaning or narrative of an experience will reduce activation in fear related areas (Hermann et al., 2014). Such reappraisal of negative experiences and memories often takes place in psychotherapy, especially cognitive and psychodynamic methods. Additionally, in logotherapy (a method created by Frankl (1992) based on his observation of people making sense of experience of extreme adversity), positive meaning is created for negative experiences, leading to less negative emotion and behavior.

## Contextual Brain and Safety Learning

*Context* plays a very important role in human behavior. Broadly, context is a set of circumstances that brings additional background information about specific cues and directs behavior. For example, the emotional response caused by exposure to a lion in the African Sahara could differ greatly from exposure to the same animal in a zoo. The physical components of context in exposure modify the natural fear response to the predator in the zoo. Context plays an important role in laboratory models of the learning of fear, and specifically its extinction. Though fear can be linked to a specific context, cue-related fear learning is independent of context (Bouton and King, 1983). This means that a person who is attacked by a dog will be afraid of dogs in any context. On the other hand, extinction learning is context-dependent. In the laboratory setting, extinction learning is best recalled in the same physical context where it took place. Therefore, return to the fear-learning context can lead to renewal of learned fear (Maren et al., 2013). This phenomenon is familiar to clinicians: exposure therapy done in the clinic does not always generalize to the original trauma context or other neutral contexts than the clinic office, which necessitates *in vivo* exposure in as many contexts as possible. Specifically in PTSD, impairment of context processing has been a recent focus of research. In this disorder, learning of fear does not differ greatly from healthy controls, but context dependent recall of extinction learning is a major impairment (Garfinkel et al., 2014).

As it was noted earlier, the broader concept of context involves perception of time, cognition, internal emotions, hormones, and physical aspects (Maren et al., 2013). Our team has previously shown that cognitive context provided in the form of instruction can function similarly to physical context in the recall of extinction learning (Javanbakht et al., 2017).

A less explored aspect of context is the perception of time. The ability to perceive time allows an organism to make sense of a sequence of events, and differentiate those of the past from those of the present. *Spontaneous recovery*, a phenomenon through which, by passage of time, a formerly extinguished fear response resurfaces, is one presentation of temporal context in fear and safety learning (Dunsmoor et al., 2015). A similar phenomenon that is commonly observed in clinic is the resurfacing of formerly treated phobias, OCD, and PTSD. To prevent this phenomenon, patients are encouraged to keep practicing *in vivo* exposure even after treatment goals are achieved. Similar to other elements of context, the processing of time is highly dependent on the hippocampus (Preston and Eichenbaum, 2013; Eichenbaum, 2013), and the anterior insula (Craig, 2009). To reiterate, both of these areas are commonly involved in extinction learning and recall, and show aberrant anatomy and function in anxiety disorders. Interestingly, the subjective experience of time can be modulated by other contextual information such as physical attributes and emotional valence (Fraisse, 1984; Noulhiane et al., 2007; Droit-Volet and Gil, 2009). In clinical practice, patients often react to the recall of a memory as if it is happening in the here and now; as if the psychic apparatus does not differentiate between “there and then,” and “here and now.” This phenomenon is often explained as “timelessness” of the unconscious in psychoanalytic theory (Scarfone, 2006). Difficulties in temporal context processing may explain fear reactions to the recall of a memory of an aversive situation, which is harmless in here and now, especially in disorders of context processing such as PTSD. One function of methods like mindfulness or meditation is training the person to bring attention from there and then, to here and now.

## Pattern Separation and Pattern Completion

Pattern separation enables a network to differentiate between two partially similar patterns, and prevent error in recall (Guzowski et al., 2004). Impairments in pattern separation are suggested to play a role in the overgeneralization of fear responses observed in fear disorders and PTSD (Kheirbek et al., 2012). In pattern completion, familiar components of a newly input pattern trigger recall of a relevant previously learned pattern to complete missing or unclear components of the new input (Rolls and Kesner, 2016). This allows accurate generalization when facing a noisy or partially known input pattern (Paleja et al., 2014). The dentate gyrus and the CA3 region of the hippocampus, with its auto-associative structure, have been the main focus of animal and human studies of pattern recognition and separation (Kolassa et al., 2010; McClelland and Goddard, 1996). Although the majority of experiments in this field are focused on spatial and visual pattern recognition, due to extensive inputs from distinct cortical areas, pattern recognition can combine perceptual, temporal, and cognitive inputs. For example, spatial inputs from the parietal lobe, and visual information from inferior temporal lobe, can enter a single hippocampal neuron (Kolassa et al., 2010). In this sense a pattern can be composed of visual and auditory components to represent a familiar person speaking, or determining a distinct language. Theoretically, prefrontal inputs to the hippocampus can present patterns of cognitive or social content. This form of pattern recognition integrates complex inputs and recognizes them as a coherent event (Barsalou, 2013). Processing the temporal component allows not only the identification of the spatial location of an object, but also its place in time (Paleja et al., 2014). Both the integration of diverse sensory information and function of pattern separation and completion contribute to contextual processing in the hippocampus. Besides generalization, pattern completion may play a role in the formation of cognitive schemas and transferences (Javanbakht, 2011; Javanbakht and Ragan, 2008). In case of transference, similar characteristics of a relational pattern (intimate, trusting, and important nature of relationship with the therapist) can trigger emotional memories, and relational patterns experienced with significant caregivers of childhood. If that caregiver was perceived as critical and judgmental, those attributes will complete what is not known about the therapist (transference), and the therapist will be perceived as judgmental. A function of the psychoanalyst is then to repeatedly present a new and adaptive pattern of relation, to help the patient encode a new relational pattern and expand the reservoir of memorized relational patterns. The empathic and understanding nature of the therapist may for long be reduced and removed as “noise” before new learning happens, which is observed in clinical practice of psychoanalysis. Furthermore, negative emotions and cognitive expectancy of a negative experience, may narrow attention to negative/threat related input (attention bias), and limit access to all of what is happening in the therapeutic context, especially positive experiences (Bar-Haim, 2010).

## Memory Reconsolidation Research

A growing body of research suggests that emotional memories may not be as solid as we once thought they were. Recent animal and human studies have shown that when memories are recalled, they become labile and vulnerable to change. In one of the first animal studies, Nader et al. (2000) showed that when fear memories are reactivated up to 14 days after fear conditioning, infusion of anisomycin in the amygdala led to amnesia of learned fear. While extinction learning involves encoding additional information to the fear memory traces indicating safety of feared cues in the new context, reconsolidation involves erasure or change of the emotional component of the fear memory. It is important to know that reconsolidation does not erase the declarative knowledge of the events, but rather the fear response to the conditioned cues (Treanor et al., 2017).

Memory reconsolidation research has led to a large amount of excitement about new ways of treating fear and anxiety related disorders, especially PTSD. While extinguished fear memories can return (clinically seen in relapse after exposure therapy), reconsolidated memories cannot. Some authors have suggested that memory reconsolidation plays an important role in psychotherapeutic process (Lane et al., 2015), while others have been more cautious (Treanor et al., 2017; Elsey et al., 2018). Evidence within memory reconsolidation research is mostly based on single cue recent fear conditioning studies. But does this apply to extremely aversive, complex traumatic memories (e.g., PTSD) repeatedly reinforced in humans? We still do not know how aversive, how complex, or how distant a memory is vulnerable to reconsolidation (Liberzon and Javanbakht, 2015). In summary, while promising, more evidence is needed to implicate memory reconsolidation research in clinical practice.

Having discussed the above processes, below I will discuss their relevance to the overlap between seemingly distinct mainstream psychotherapeutic approaches to treating fear and anxiety related disorders.

## Behavioral Therapy

Behavioral therapy, which is based on principles of associative learning of fear and its extinction, is one of the most commonly used treatments for fear related disorders, OCD, and trauma (Newman, 2016). In behavioral therapy, the patient is exposed to a feared cue or situation, in a safe context. After repeated exposure, extinction learning occurs and the cue will no longer trigger a fear response. This method is used for a diverse array of anxiety disorders, where there is an internal or external cue or situation that is feared. In phobias, the cue is an external perceived object or situation. In PTSD, exposure is to autobiographical memories, and overly generalized fear response to safe cues. In OCD and nightmare disorders, exposure is to autobiographical memories and cognitive constructs. As in extinction learning, contextualization plays an important role in exposure therapy. Commonly in clinical practice it is observed that the safety learned in a clinic setting may not apply to real life conditions. Similarly, return to the context of trauma (e.g., the parking lot where the assault happened), may lead to *renewal* of the fear response. Furthermore, because extinction learning is not an erasure of the learned fear, even after successful therapy, fear responses may return (Milad and Quirk, 2012). For all these reasons, patients are encouraged to continue *in vivo* exposure in as many contexts and with as many cues possible, even after completion of treatment, to prevent such *renewal*, or *spontaneous recovery* of the fear response.

An important element of exposure that is often overlooked in laboratory models is the use of the therapist as a social safety cue and as an anchor in time. The therapist is a continuous reminder to the patient that they are in a different temporal, physical, and social context than the time trauma happened and fear was learned. In the disorders where fear is linked with an autobiographical intrusive memory (OCD, PTSD, nightmare), the therapist’s communication with the patient frequently brings them back from “there and then” to the “here and now,” which facilitates the process of contextualization of fear memories. In other words, the therapist helps the patient to put those memories back in their time and place. The therapist also provides a sense of safety (social learning of safety), and enforces a sense of control in the patient. This sense of control is pivotal as the person chooses to encounter the feared situation, rather than being surprised by it.

In summary, cue generalization, safe social cue, sense of control, and contextualization seem to be the most important elements of successful exposure therapy.

## Cognitive Therapy

Cognitive therapy is commonly used in the treatment of anxiety disorders, especially those with a larger component of anxiety than fear, such as GAD, and is often used in combination with exposure therapy. In such therapy, cognitive constructs that trigger negative emotions are addressed and challenged, and pros and cons of such beliefs are discussed with the patient, and then replaced by more adaptive and realistic patterns. In that sense, reappraisal plays a role in cognitive therapy, as often times the meaning and interpretation of the experiences are what changes during this process.

The process of cognitive therapy may also include some level of associative learning, linking emotions to complicated cognitive constructs rather than simple cues. For example, in the red car example explained earlier, perception of a young person driving a red car, can trigger the combination “young person, driving, red, car = threat” which is linked with a fear response. During cognitive therapy, this cognitive compound is challenged and modified to a more adaptive one. When maladaptive cognitive constructs are recalled, there is an opportunity for a new associative learning of safety to take place. In other words, frequent exposure to the cognitive construct happens in presence of a safe social cue (therapist), who challenges the emotional response, and prevents avoidance, leading to safety learning. In this sense, exposure and cognitive therapy overlap not only in method, but also in mechanism. In the first method the focus is on safety learning for an external object or autobiographical memory, and in the latter, it is a cognitive construct, and related real life experiences.

Similar to exposure therapy, the therapist’s role (other than offering new explanations to enrich the schematic patterns of interpretation and make sense of events) is to enforce contextualization, and provide a sense of control and safety. During exposure therapy the patient experiences safety near a feared object with the therapist, while in cognitive therapy the patient experiences safety in exposure to a feared cognitive construct. Temporal contextualization brings the patient to here and now, and away from the possible threat environment of the past where the distorted cognitive constructs formed.

## Psychoanalysis and Psychodynamic Therapy

Traditional psychoanalysis is mainly composed of transference interpretation and free association. Transferences are emotional patterns formed towards significant persons of the past, and are transferred onto the therapist in the context of treatment. During free association, patients share their automatic flow of thoughts in response to the discussed events, memories, and experiences without the therapist’s influence (Tuch, 2017). Then the two will try to make sense of this stream of thoughts. Another important element of psychoanalysis and psychodynamic theory (the less traditional form of therapy rooted in psychoanalysis) are defense mechanisms, introduced by Anna Freud. Defense mechanisms are thought to be automatic unconscious processes developed to avoid anxiety and conflict stirred by internal or external experiences (Freud, 1967). The underlying mechanism of this defense is still to be explained (Northoff et al., 2007).

Although on the surface, psychoanalysis seems to be the most distant method from behavioral therapy, there may be similarities in mechanisms. As was noted earlier, transference formation can be explained as associative learning involving complex cognitive or interpersonal patterns, and overgeneralization of such associative learning. For instance, childhood exposure to a hypercritical parent may link that parent to experience of fear, insecurity, or anger. Alternatively, the fear may become associated with the parent’s role as an authority, and be generalized to relationship patterns with other authority, intimate, or important figures. This could apply to any other component of the parent, e.g., their gender (e.g., maternal transferences toward significant female persons). Consequently, the context of the therapeutic relationship can trigger recall of relevant autobiographical memories or implicit cognitive constructs related to a parental figure, leading to a fear or anger response. In this context, similar to exposure therapy, one mechanism of therapy work is frequent exposure to the feared perceived object of authority/parental figure, or other relationship patterns. When the transferences are repeatedly experienced without an aversive critical response from an empathic therapist, extinction learning occurs and the new relational pattern is added to the memory reservoir. This process in psychoanalysis is referred to as *corrective emotional experience*, “to re-expose the patient, under more favorable circumstances, to emotional situations which he could not handle in the past. The patient, in order to be helped, must undergo a corrective emotional experience suitable to repair the traumatic influence of previous experiences” (Alexander et al., 1946).

In psychoanalysis, implicit associative learning may also include experience of an emotion. For instance, a person who was often punished when having fun during childhood, learns that the experience of joy, or internal context of positive emotions predict threat/pain. Consequently, the experience of joy and pleasure-related contexts would trigger fear or sadness without an explicit awareness of the association. A patient of mine complained about drinking too much in social contexts, and then embarrassing herself. At further exploration, she identified an automatic thought that she is only accepted and perceived as fun when she is drunk. Later on, she remembered that during her childhood, home was a sad place, but she always found a happy respite at the Smith’s home (the neighbors). She explained that they were “the only people who paid attention to me.” The Smith couple was always drunk when she visited them, and the patient realized later that she associated drinking, and even the smell of alcohol, to experience of being loved and accepted. Although the formulation is a psychodynamic one, the cognitive distortion, and the associative learning are evident in this example. For this patient, the scent of alcohol, social and sensory cues related to alcohol, triggered associated memories of feeling safe and happy, and being loved and accepted.

Becoming aware of the underlying associative memories that trigger automatic emotional responses is an overlapping function of psychoanalysis and cognitive therapy. While in cognitive therapy, awareness is directed toward automatic distorted cognitive constructs that trigger maladaptive emotional response, in psychoanalysis, awareness is of the autobiographical memories, both cognitive and emotional, which are automatically generalized to a range of internal and external contexts and cues. In both methods of therapy, besides bringing implicit functions to awareness, reappraisal and development of a different meaning for the same experience is a key element.

Defense mechanisms are used in clinical practice even by clinicians who do not use other psychodynamic principles such as transference and free association. Defense mechanisms are functions of the “ego” that serve the purpose of avoiding anxiety provoking or conflicting thoughts, memories, or impulses (Freud, 1967). Among the most common defense mechanisms are projection (when one’s own thoughts or emotions are attributed to others to avoid acknowledging their presence in oneself), displacement (when thoughts or impulses are displaced to a safer object, e.g., yelling at the dog because it is safer than yelling at the boss), and denial (of anxiety provoking stimulus, thought, or impulse). Although defense mechanisms are commonly used in clinical practice to understand behavior, the underlying mechanism is unclear and they have yet to be explained in the context of modern neuroscience.

A defense mechanism is not always a planned process to avoid conflict or emotional pain. An example of displacement is when a person is angry with their boss, but releases it at home. At the workplace, other contextual (work related social context), cognitive (if I yell at authorities, I will get hurt, or fired), and autobiographical or factual memories (my friend got fired because he talked back to his boss) prevent the person from acting out an anger impulse. When at home, the internal context of anger, and anger related behavioral and relational patterns are still present. Shifted attention towards negative external cues, will enforce perception of what the dog is doing wrong, and yelling at the dog is less costly. In this sense, yelling at the dog is not replacing the boss, but is triggered by it. Reduction in functional connectivity between emotion regulatory, cognitive, and contextual processing prefrontal cortices, and amygdala may explain the defense mechanism of denial/absence of conscious awareness of highly conflicting autobiographical memories or perceptions (Birn et al., 2014; Bijsterbosch et al., 2015). In this case, the emotion is not removed from awareness to protect the ego against anxiety, but rather is not experienced simply because of reduced connectivity. Similarly, projection defense may result from attention bias and pattern completion of external stimuli due to internal emotional and cognitive contexts (Javanbakht and Ragan, 2008).

Similar to cognitive and behavioral therapy, the therapist has a pivotal role as a safe social cue in learning of safety, and in helping the patient in physical, social, and temporal contextualization. The analysand moves back and forth between the context of old autobiographical memories and associated implicit and explicit emotions, to therapist, who is anchored in the here and now physical, interpersonal, and temporal contexts.

It is important to note that psychoanalysis is a complex theory with a variety of facets and implications. For instance, this theory has important contribution to personality and its disorders, which is beyond the scope of this work. Here I only address psychoanalysis and its use in anxiety related disorders and trauma.

## Psychotropic Medications and Psychotherapy

Currently, medications used for the treatment of all fear and anxiety related disorders remain the same: serotonin specific reuptake inhibitors, serotonin and norepinephrine reuptake inhibitors, and at times benzodiazepines. These medications seem to work by reducing the phasic and tonic level of arousal, anxiety, and the phasic fear response. They can reduce the amygdala’s response to negative stimuli, and increase prefrontal emotion regulation (McCabe et al., 2010; Outhred et al., 2013). Reduced baseline and reactive anxiety can modulate threat-oriented biased attention, and recall of negative memories, signal a safer internal context, and facilitated more realistic pattern recognition and contextualization of the input information.

Based on the context in which they are received, the relevant cognitive schemas and emotions linked to the experience of taking medication, drugs may trigger different emotional and cognitive patterns. For instance, while in one patient taking medication may implicitly trigger associated cognitive and autobiographical components of “mom, Fluoxetine, hospital, angry dad,” in another patient the associated memories may include “mom, medication, happy, vacation,” and yet in a third person they may be “green pills, girlfriend, conflict, suicide.” While in the first patient the cue “anxiety medication” can trigger emotions of fear, despair, or anger, in the second patient it may trigger hopeful feeling of relief, and in the third patient disappointment and guilt. This function of the medication in triggering autobiographical and emotional memories, associative learning, and cognitive schemas, may explain placebo effects, the unpredictable level of effect across patients, and side effect variability despite their similar mechanism of action, and their unexpected quick effects in some. Despite the fact that SSRI medications require several weeks to start benefiting the patient, reports of quick effects even in less than a week are common in clinical practice.

From the psychoanalytic standpoint, medication may work as a *transitional object*, first described by Winnicott (1969). A common example of a transitional object is the teddy bear that represents mother’s presence, and allows the child to wander away from mother, while carrying a piece of her. Medication can operate as a transitional object for the treating physician, and taking it can evoke emotional, cognitive, and social patterns of perception of the physician, and those transferred onto the treatment relationship.

Recent research has been done on pharmacological agents as enhancers of extinction learning, or disrupters of reconsolidation of aversive memories. While several agents are used for these purposes in laboratory research of single cue conditioned memories, there is limited clinical evidence for a few of these agents. A few studies have shown small effects for the partial NMDA agonist D-Cyclocerin, SSRI’s, and endocannabinoids as enhancer of exposure therapy (For reviews see Fitzgerald et al., 2014; Mataix-Cols et al., 2017). The beta-adrenergic blocker propranolol has been suggested as a disruptor of reconsolidation of traumatic memories (Gardner and Griffiths, 2014; Giustino et al., 2016). In a recent promising study, use of propranolol 90 min before reactivation of traumatic memories in PTSD patients for 6 weeks, led to larger decline in symptoms severity than placebo (Brunet et al., 2018). Authors suggest this effect is through disruption of reconsolidation of recalled and labile memories. Another function of propranolol may be reduction of adrenergic arousal level, leading to a calmer internal context while the memories are recalled. This may help in dissociating the traumatic memory from the extremely aversive emotional tone, and helping in contextualization of the memories in the safe here and now.

## Conclusion and Clinical Implications

As our understanding of the neurobiological mechanisms of formation and regulation of fear has evolved, we seem to find not only overlapping clinical use, but also neuronal mechanisms for seemingly distinct methods of therapy used for the same psychopathologies. In this work, I proposed some of these common mechanisms including associative learning of safety and extinction learning, its generalization, and contextualization. As our laboratory understanding of the concept of context has evolved, more complicated aspects of cognitive, internal, social, and temporal contexts seem to play a role in contextualization of safety learning in clinical setting. Cognitive reappraisal, and modification of meaning of experiences is another important component of different therapies. Finally, the therapist seems to play a critical role as a social cue in safety learning, an anchor in the here and now, that promotes social, physical, and temporal contextualization of memories of the past.

Maslow (1966) once wrote: “If the only tool you have is a hammer, every problem begins to resemble a nail.” The possibility that different methods of therapy have overlapping mechanisms encourages utilizing these methods in combination. It seems reasonable to thoughtfully utilize principles of the seemingly distinct therapies to increase and expedite the outcome, rather than orthodoxy in using only one method. For example, while interpretation of transferences in psychoanalysis helps in development of conscious insight to the common patterns of perceiving self and others, extinction learning may be a mechanism in reducing fear response to perception of these patterns. Consequently, besides the traditional approach of psychoanalysis, it seems reasonable to encourage *in vivo* exposure to those patterns to foster generalization and contextualization of safety learning. In psychodynamic therapy, if the concept of “authority” is disentangled from threat, then this new learning of safety can more easily generalize to other conditions (Bieber, 1980). On the other hand, in exposure-based therapies, a psychodynamic understanding of the broader patterns of fear generalization will help in development of broader approaches to the feared object category, and prevent future emergence of the fear response to similar patterns. This fluid exchange of mechanism and execution could potentially create a far more effective intervention than any one theory alone could provide.

Similarities of mechanism in therapies may also suggest a possibility of more efficient approaches to psychoanalysis and psychodynamic therapy. Traditional psychoanalysis often involves years of intense treatment with several sessions a week, which is not affordable for most patients. However, there is evidence for use of psychodynamic treatment with lesser density and shorter time periods (Kernberg, 2015). If indeed some of the same mechanisms of extinction learning and reappraisal are involved in psychodynamic therapy, then briefer methods may reasonably work in shorter lengths of time. Furthermore, the idea of a therapist as a blank screen introduced by Freud (rather an obsolete concept in modern psychoanalysis) should perhaps be replaced by a more empathic and involved therapist, which will play a better role as a social safety cue in learning of safety.

An old debate (especially in psychoanalysis) surrounds the efficacy of combining medications and psychotherapy, or therapist and prescriber, some suggesting their separation (Cabaniss, 2001). A combined approach may seem more reasonable, keeping in mind that medications can help in the process of therapy, by reducing the anxiety level within the optimal learning window. Extremely high levels of anxiety may reduce the amygdala’s connectivity with brain’s emotion regulatory areas, general cognition, and the patient’s ability to be involved in therapy. If exploration into the use of memory modulating agents is proven successful by research, then the argument for integration may be furthered (Singewald et al., 2015). On the other hand, overuse of medications may impair learning by reducing the arousal level below the optimal learning window, or by simply impairing learning, in case of benzodiazepines use (Tyng et al., 2017). Finally, since medication plays a broader psychological role beyond the pharmacological agent affecting neurotransmission, it seems reasonable to be discussed in the process of therapy.

The use of technology in psychiatry and psychotherapy has been emerging in recent years. Telepsychiatry has brought providers to patients’ homes and is a rapidly growing field with similar efficacy to office treatment, even used by psychoanalysts (Hilty et al., 2015). Telepsychiatry can also provide therapists with the opportunity of an *in vivo* contextualization. Additionally, virtual reality methods have enabled therapists to bring more exposure scenarios to the clinic, and advance cue generalization by providing a diverse number of feared objects (Opriş et al., 2012). The newest technology, augmented reality, offers the ability to overlay virtual objects onto real life physical contexts. This technology, combined with telepsychiatry, can provide us with the unique opportunity of connecting therapist and patient in their real life context, and adding a diverse range of feared objects to the *in vivo* context. This way, the social safety cue, cue generalization, and contextualization may all happen at the same time and place!

## Author Contributions

The author confirms being the sole contributor of this work and has approved it for publication.

## Conflict of Interest Statement

The author declares that the research was conducted in the absence of any commercial or financial relationships that could be construed as a potential conflict of interest.
